# The Effect of Postoperative Corticosteroid Administration on Free Vascularized Fibular Grafting for Treating Osteonecrosis of the Femoral Head

**DOI:** 10.1155/2013/708014

**Published:** 2013-11-12

**Authors:** Hao Ding, Sheng-Bao Chen, Sen Lin, You-Shui Gao, Chang-Qing Zhang

**Affiliations:** Department of Orthopedic Surgery, Shanghai Sixth People's Hospital, Jiao Tong University, Shanghai 200233, China

## Abstract

Free vascularized fibular grafting (FVFG) has been reported to be an effective method of treating osteonecrosis of the femoral head (ONFH). This study evaluated whether postoperative maintenance doses of corticosteroids had an adverse effect on FVFG outcomes in patients with corticosteroid-induced ONFH. We retrospectively reviewed the records of 39 patients (67 hips) who had received maintenance doses of corticosteroids following FVFG. This group was matched to a group of patients who had not received corticosteroids treatment after operation. The mean follow-up duration was 5.4 years for the postoperative corticosteroid administration group (PCA group) and 5.0 years for the control group. At the latest follow-up, the average increase in Harris hip score was 11.1 ± 8.7 points for all hips in the PCA group and 12.6 ± 7.4 points for all hips in the control group (*P* > 0.05). In the PCA group, through radiographic evaluation, 49 hips were improved, 10 hips appeared unchanged, and 8 hips appeared worse. In the control group, 47 hips were improved, 13 hips appeared unchanged, and 7 hips appeared worse. The results suggested that postoperative maintenance doses of corticosteroids do not have an adverse effect on FVFG outcomes in patients with corticosteroid-induced ONFH.

## 1. Introduction

Corticosteroid-induced osteonecrosis of the femoral head (ONFH) is a serious complication of systemic corticosteroid administration for treatment of autoimmune diseases such as systemic lupus erythematosus (SLE), nephrotic syndrome, and rheumatoid arthritis. The prevalence of ONFH in patients receiving corticosteroids has been reported as 0.3~13% [1, 2]. Most patients with ONFH require surgical treatment for pain relief and improvement of hip joint function. Current treatment options for ONFH include conservative treatment, core decompression, vascularized bone grafting, and total hip arthroplasty.

Generally, patients with autoimmune diseases receive high-dose corticosteroids early in the course of the disease. The corticosteroid dose of these patients is subsequently gradually decreased to a maintenance dose (daily prednisolone-equivalent dose is 10 mg or below) as clinical improvement is achieved. Maintenance doses of corticosteroids can be given for three months to several years. For some patients, ONFH was first recognized when they were receiving maintenance doses of corticosteroids for their autoimmune diseases. Most of these patients should receive operation immediately, because it is easier to get the satisfactory prognosis at the early stage of ONFH [3, 4]. After operation, maintaining continuous treatment of the primary disease does not permit cessation of corticosteroid in most of them. However, corticosteroids have harmful impact on the femoral head at many aspects. Takano-Murakami et al. found that supraphysiologic doses of glucocorticoids suppressed osteoblast proliferation and the recruitment of osteoclast precursors [5]. Corticosteroids also promote bone marrow stromal cells to develop into adipocytes, while increasing the size of fat cells [6, 7]. Drescher et al. believed that methylprednisolone enhances contraction of the femoral head's lateral epiphyseal arteries and reduces femoral head blood flow [8]. In this case, the adverse impact of corticosteroids on the femoral head might still exist after operation. It is widely accepted that high dose of corticosteroids can lead to ONFH. However, there are no reports on whether maintenance doses of corticosteroids affect operation outcomes in patients with corticosteroid-induced ONFH.

Free vascularized fibular grafting (FVFG) is an effective method of halting progression of osteonecrosis and promoting bone regeneration at necrotic foci and provides good outcomes for patients with ONFH at early stage [9, 10]. This study was performed to compare the FVFG outcomes in patients who had received maintenance doses of corticosteroids with patients who had not received corticosteroids treatment after FVFG and determine the effect of postoperative corticosteroids on FVFG outcomes.

## 2. Materials and Methods

### 2.1. Patient Selection

We retrospectively reviewed the records of patients with corticosteroid-induced ONFH who received FVFG in our hospital from 2000 to 2010. Diagnoses of ONFH were based on history, clinical evaluation, and imaging modalities including anteroposterior and frog-leg lateral radiographs as well as magnetic resonance imaging (MRI). The Steinberg classification was used to evaluate radiographs, and ONFH was classified by stages from 0 to VI [11]. Patients with stages II, III, and IV of ONFH were performed with FVFG. Before surgery, patients with corticosteroid-induced ONFH were evaluated by practicing physicians to ensure that primary diseases were under control and that the daily dose of corticosteroids was 10 mg or less.

Inclusion criteria for the postoperative corticosteroid administration group (PCA group) was ongoing maintenance corticosteroid therapy for the primary disease for at least 6 months after the FVFG. The follow-up periods were at least two years. Patients with deterioration of the primary disease or those whose corticosteroids dose had exceeded 10 mg/day during the follow-up period were excluded. The PCA group was matched to a group of patients with corticosteroid-induced ONFH who had not received corticosteroids treatment after FVFG. Matching was based on gender, average age, preoperative corticosteroid dose, preoperative Steinberg stage, and preoperative Harris hip score (HHS).

### 2.2. Preoperative Evaluation

Preoperative assessments, including complete blood cell counts, erythrocyte sedimentation rates, C-reactive protein assays, urea monitoring, and electrocardiography were performed as appropriate to ensure patient fitness for surgery. Patient demographic characteristics and information regarding corticosteroid administration (route, daily dose, total cumulative dose, and duration of corticosteroid treatment) were recorded. When multiple corticosteroids were used, an equivalent dose of prednisolone was calculated as a standard for comparison. Clinical and imaging data that were recorded included Harris hip score (HHS), plain radiographs, and MRI.

### 2.3. Operative Management

All surgeries were performed by the corresponding author using previously reported methods [12]. During surgery, histological examination of subchondral bone was performed to confirm the diagnosis of ONFH. Postoperative prophylactic antibiotics were used twice a day for three days, and anticoagulants were administrated for six weeks after the operation. Postoperative pain was managed by administering NSAIDs. Patients were instructed to avoid bearing weight on the leg that received the FVFG for three months, followed by gradually increased weight bearing to full weight bearing over the following three months.

### 2.4. Follow-Up

Follow-up examinations were performed every 3 months for 1 year, every 6 months for 3 years, and annually thereafter. The end point was conversion to a total hip replacement. During the follow-up period, clinical and radiographic results, information about postoperative steroid administration, and postoperative complications were recorded.

Clinical results were evaluated using HHS. They were considered excellent for HHS ≥ 90 points, good for HHS 80–89 points, fair for HHS 70–79 points, and poor for HHS < 70 points. Radiographic evaluations were independently performed by two radiologists that were blind to the clinical results. Femoral heads were postoperatively assigned to one of three categories, based on radiography. (1) Improved: that is, the necrosis was healed or was being replaced with new bone. In stage II, the crescent had disappeared or the density of cystic lesion had increased with trabecular formation at the tip of the vascularized fibula. In stage III, the collapsed lesion healed or became more rounded with trabecular formation at the tip of the vascularized fibula. (2) Unchanged: that is, no change or no progression occured, compared to the preoperative state. (3) Worse: that is, necrosis progressed, based on the stage or where the femoral head collapsed by more than 3 mm.

### 2.5. Statistical Analysis

Statistical analysis was performed using the SPSS 17.0 statistical package (SPSS, Chicago, IL, USA). Two-sample K-S tests were used to compare values in the PCA group and the control group. A *P* value less than 0.05 was considered to be statistically significant. The study was approved by the Regional Ethics Committee.

## 3. Results 

### 3.1. General Conditions

Thirty-nine patients (67 hips) met the inclusion criteria for the PCA group. Demographic characteristics and corticosteroid administration of patients in the PCA group and the control group were summarized in Table 1. All patients were ethnic Chinese. Primary diseases requiring corticosteroid treatment included SLE, renal diseases, idiopathic thrombocytopenic purpura, and dermatomyositis. No patients in the two groups had serious acute complications. One patient in the PCA group had deep vein thrombosis and was successfully treated with oral medication. Three limbs in the PCA group and two limbs in the control group appeared clawing of the big toe, were treated nonsurgically, and recovered gradually. Two hips in the PCA group and three hips in the control group had wound hematomas. Four patients (4 hips) in each group received total hip arthroplasty (THA) during the follow-up, and their scores immediately before THA were included as their final scores. All patients in the two groups fully complied with rehabilitation instructions.

### 3.2. Clinical Results

Harris hip scores were used to evaluate clinical outcomes of the patients. In the PCA group, the mean preoperative HHS was 72.0 ± 9.1 points for all hips and the mean postoperative HHS was 83.2 ± 10.9 points. In the control group, the mean preoperative HHS was 72.3 ± 8.5 points and the mean postoperative HHS was 84.9 ± 10.2 points (Table 2). The average increase in scores was 11.1 ± 8.7 points for all hips in the PCA group and 12.6 ± 7.4 points for all hips in the control group. According to the Steinberg classification, the average increase in scores was 10.7 ± 6.0 points for stage II hips in the PCA group, 11.7 ± 5.7 points for stage II hips in the control group; 11.7 ± 8.6 points for stage III hips in the PCA group, 14.2 ± 6.0 points for stage III hips in the control group; 10.7 ± 10.6 points for stage IV hips in the PCA group, 11.4 ± 9.6 points for stage IV hips in the control group. There was no significant difference between them (*P* > 0.05).

### 3.3. Radiographic Results

**Figure 1:**
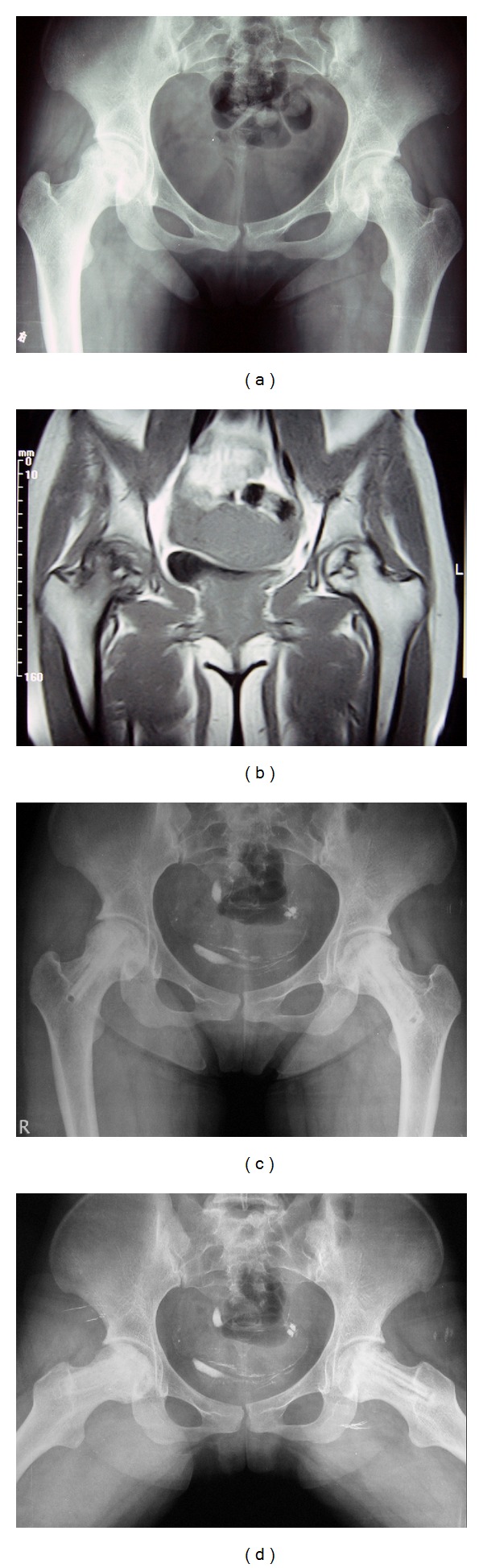
(a) Preoperative radiographs (anteroposterior position of the pelvis) of a 22-year-old woman with Lupus and stage II bilateral hip osteonecrosis. The preoperative Harris hip scores were 74 (right) and 85 (left). The patient received corticosteroid treatment at maintenance doses until the latest follow-up. (b) Preoperative MRI of this patient. (c) and (d) Postoperative radiographs (anteroposterior position and frog-leg position) taken three years after FVFG showed stability of the articular cartilage and femoral head. The postoperative Harris hip scores at the latest follow-up were 90 (right) and 96 (left).

Radiographic results were evaluated using the criteria described above and summarised in Table 3. In the PCA group, 49 hips were improved in the last radiographs (Figure 1), 10 hips appeared unchanged, and 8 hips appeared worse. In the control group, 47 hips were improved, 13 hips appeared unchanged, and 7 hips appeared worse.

## 4. Discussion

The current operations to treat ONFH include core decompression, proximal femoral osteotomy, vascularized bone grafting, and total hip arthroplasty. For many young patients, hip replacement cannot be expected to last the patient's lifetime. Therefore, attempts should be made to save the femoral head prior to collapse. Conservative treatment is only recommended for patients in preliminary stages of ONFH when symptoms are not significant. The outcome is usually poor with this option, due to the natural history of ONFH [13]. Core decompression is a less invasive surgery and theoretically interrupts the process of ONFH to heal the femoral head [14]. However, conflicting clinical results and the variable natural history of ONFH make interpretation of these studies difficult. The osteotomy was reported to have favourable results in treating ONFH [15]. However, the success of the osteotomy is related to the extent and location of the necrotic lesions. The complication of the osteotomy, such as shortening of the leg and gait abnormalities, remains a concern [16].

Free vascularised fibular grafting is an appealing alternative to core decompression for treatment of ONFH, especially for patients with subchondral bone collapse. During the procedure, the necrotic bone is excised, which may interrupt the cycle of ischaemia and intraosseous hypertension and promote local revascularisation. Then the defect is filled with osteoinductive graft to support the subchondral surface. After the surgery, a period of limited weightbearing benefits the healing construct. Yoo et al. reviewed 81 hips that received FVFG, with a mean follow-up period of 5.2 years and found that 71% had radiological improvement [17]. Judet and Gilbert assessed 68 hips with an average follow-up of 18 years, and found that good results were achieved in 80% of the patients [18]. Berend et al. found that patients with postcollapse osteonecrosis of the femoral head benefit from FVFG, with good overall survival of the joint [19].

In this study, we evaluate the effect of postoperative maintenance doses of corticosteroids on FVFG outcomes. Two groups of patients were matched as closely as possible according to gender, average age, preoperative corticosteroid dose, preoperative Steinberg stage, and preoperative Harris hip score (HHS), which may be important variables for the prognosis of ONFH. Harris hip score and radiographic changes were used to assess the outcomes in the patients of two groups. The results showed obvious increase in HHS in both groups and there was no significant difference between them. In addition, most of the radiographic results were improved in both groups. Therefore, we believed that postoperative maintenance doses of corticosteroids did not have an adverse effect on the outcomes of FVFG for treatment of corticosteroid-induced ONFH. There is no need to stop corticosteroids treatment to aid the healing of ONFH after the surgery.

There may be two potential reasons for the results. First, the postoperative steroid doses were low. Studies found that corticosteroids adversely impact the femoral head in a dose-dependent manner [20–22]. Maintenance doses of corticosteroids may minimally affect the recovery of ONFH. Second, FVFG has an excellent ability to promote new bone regeneration and revascularization to an extent that exceeds the harmful effects of low-dose corticosteroids. However, we are aware of our study's limitations. First, the current study has only 39 patients, and rigorous conclusions are difficult. Second, the length of follow-up is about 5 years. Therefore, longer-term research with larger numbers of patients will be performed to further confirm the conclusion in the future.

## Figures and Tables

**Table 1:** Demographic details of patients.

Variables	PCA group	Control group
Age at the time of surgery (y)	28.6 (17–38)	29.1 (18–38)
Female : male (*n*)	30 : 9	28 : 11
Cumulative prednisolone dose before surgery (g)	10.4 (5.2–19.1)	11.1 (4.3–21.2)
Preoperative Steinberg stage (hips)		
Stage II	19	19
Stage III	25	25
Stage IV	23	23
Preoperative Harris hip score	72.0 ± 9.1	72.3 ± 8.5
Stage II	80.4 ± 6.2	81.4 ± 5.7
Stage III	72.9 ± 6.1	72.3 ± 4.5
Stage IV	64.4 ± 7.5	65.2 ± 6.8
Mean daily dose of prednisolone after surgery (mg)	5.6 (2.5–10)	0
Duration of corticosteroid treatment after surgery (mo)	28.6 (9–66)	0
Mean follow-up (y)	5.4 (2–10)	5.0 (2–10)

**Table 2:** Preoperative and postoperative Harris hip scores (HHS) in the postoperative corticosteroid administration group (PCA group) and the control group.

HHS	Total series		Stage II		Stage III		Stage IV	
	Preoperative	Postoperative	Preoperative	Postoperative	Preoperative	Postoperative	Preoperative	Postoperative
PCA group	72.0 ± 9.1	83.2 ± 10.9	80.4 ± 6.2	91.4 ± 6.8	72.9 ± 6.1	84.7 ± 8.3	64.4 ± 7.5	75.1 ± 10.7
Control group	72.3 ± 8.5	84.9 ± 10.2	81.4 ± 5.7	93.1 ± 6.8	72.3 ± 4.5	86.6 ± 7.1	65.2 ± 6.8	76.6 ± 9.3

**Table 3:** Radiographic results in the postoperative corticosteroid administration group (PCA group) and the control group.

Outcome	Stage II		Stage III		Stage IV	
(hips)	PCA	Control	PCA	Control	PCA	Control
Improved	16	15	18	20	15	12
Unchanged	2	3	4	3	4	7
Worse	1	1	3	2	4	4
